# Supplementary material for: Characterization of Structural and Energetic Differences between Conformations of the SARS-CoV-2 Spike Protein
Source: Materials (Basel). 2020 Nov 26;13(23):5362. doi: 10.3390/ma13235362 (PMC7730245; doi:10.3390/ma13235362)
Supplement: Supplementary file 1 [file materials-13-05362-s001.pdf]

materials MDPI

Supplementary Materials

Characterization of Structural and Energetic Differences between Conformations of the SARS-CoV-2 Spike Protein

Rodrigo A. Moreira [1], Horacio V. Guzman [2], Subramanian Boopathi [3], Joseph L. Baker [4] and Adolfo B. Poma [1,*]

1 Department of Biosystems and Soft Matter, Institute of Fundamental Technological Research, Polish Academy of Sciences, Pawińskiego 5B, 02-106 Warsaw, Poland; rams@ippt.pan.pl
2 Department of Theoretical Physics, Jožef Stefan Institute, Jamova 39, 1000 Ljubljana, Slovenia; horacio.guzman@ijs.si
3 Instituto de Ciencias Físicas, Universidad Nacional Autónoma de México, Cuernavaca 62210, Mexico; boopathialzheimer@outlook.com
4 Department of Chemistry, The College of New Jersey, 2000 Pennington Road, Ewing, NJ 08628, USA; bakerj@tcnj.edu
* Correspondence: apoma@ippt.pan.pl

Table S1. Number of intrachain (AA, BB, CC) and interchain (AB, AC, BC) contacts, and respective residues, from the differential contact map (dCM > 0.9) analysis using high-frequency contacts. Amino acids are classified as Polar (Q, N, T, S), Hydrophobic (A, V, I, L, M, F, Y, W), Charged (R, K, D, E) and Other (H, C, U, G, P). Stabilizing/Destabilizing contacts and residues are shown in Figures 2 and 3. The relative stability of spike protein with one and two open RBD are compared to the closed conformation and compared between themselves.

**Chains**	**Contacts**											**Residues**				
	**TOTAL**	**P-P**	**P-H**	**P-C**	**P-O**	**H-H**	**H-C**	**O-H**	**C-C**	**O-C**	**O-O**	**TOTAL**	**P**	**H**	**C**	**O**
1up2down stabilized compared to 3down																
AA	3	0	1	0	1	1	0	0	0	0	0	6	2	3	0	1
BB	24	2	3	0	1	4	5	3	2	4	0	32	7	9	11	5
CC	11	1	0	0	4	3	1	2	0	0	0	14	4	5	1	4
AB	0	0	0	0	0	0	0	0	0	0	0	0	0	0	0	0
AC	0	0	0	0	0	0	0	0	0	0	0	0	0	0	0	0
BC	0	0	0	0	0	0	0	0	0	0	0	0	0	0	0	0
1up2down destabilized compared to 3down																
AA	9	1	1	0	0	2	1	2	1	1	0	17	3	7	4	3
BB	26	1	5	0	1	3	3	9	2	0	2	37	7	15	6	9
CC	3	0	0	0	0	0	1	2	0	0	0	6	0	3	1	2
AB	10	0	0	3	0	0	3	3	0	1	0	15	2	5	5	3
AC	0	0	0	0	0	0	0	0	0	0	0	0	0	0	0	0
BC	16	1	5	4	0	2	2	1	0	1	0	19	6	8	4	1
2up1down stabilized compared to 3down																
AA	9	0	1	0	1	4	0	2	0	1	0	18	2	11	1	4
BB	32	3	3	1	3	6	3	5	3	5	0	47	11	15	12	9
CC	13	1	1	1	3	4	0	3	0	0	0	17	6	6	1	4
AB	19	0	2	2	0	7	1	5	1	1	0	21	3	10	3	5

AC	2	1	0	0	0	1	0	0	0	0	0	4	2	2	0	0
BC	1	0	0	0	0	0	0	0	0	1	0	2	0	0	1	1
2up1down destabilized compared to 3down																
AA	18	0	6	3	0	6	3	0	0	0	0	29	8	17	4	0
BB	61	4	10	3	4	7	8	20	1	0	4	75	19	31	9	16
CC	10	0	1	1	1	2	4	1	0	0	0	16	3	7	4	2
AB	20	0	3	4	0	0	4	4	1	3	1	26	5	8	6	7
AC	4	0	0	2	0	0	2	0	0	0	0	6	1	2	3	0
BC	24	1	6	7	1	3	3	1	1	1	0	21	7	7	5	2
2up1down stabilized compared to 1up2down																
AA	6	0	1	0	0	3	1	0	0	1	0	12	1	8	2	1
BB	3	0	0	0	2	0	0	0	0	1	0	6	2	0	1	3
CC	2	0	0	0	0	0	0	2	0	0	0	4	0	2	0	2
AB	20	0	2	3	0	7	1	5	1	1	0	21	3	10	3	5
AC	0	0	0	0	0	0	0	0	0	0	0	0	0	0	0	0
BC	0	0	0	0	0	0	0	0	0	0	0	0	0	0	0	0
2up1down destabilized compared to 1up2down																
AA	15	0	4	1	1	5	3	1	0	0	0	22	3	14	3	2
BB	18	0	1	0	1	3	4	7	0	0	2	22	2	11	3	6
CC	2	0	1	0	0	0	1	0	0	0	0	4	1	2	1	0
AB	2	0	0	0	0	0	0	1	0	1	0	4	0	1	1	2
AC	10	0	1	2	0	1	3	2	0	1	0	11	1	4	4	2
BC	0	0	0	0	0	0	0	0	0	0	0	0	0	0	0	0

Table S2. List of 10 high-frequency contacts involved in detachment of the RBD in chain B from S2 domain in chain A mediated by hinge domain. Value of the dCM analysis is given in the last column.

Residue 1 RBD	Residue2 S2	dCM (freq >0.9)
S383(B)	R983(A)	1
V382(B)	R983(A)	1
S383(B)	D985(A)	1
K386(B)	S982(A)	0.97
G381(B)	R983(A)	0.96
Y396(B)	P230(A)	0.94
K386(B)	L984(A)	0.94
G381(B)	L984(A)	0.91
K386(B)	R983(A)	0.91
V382(B)	L984(A)	0.9

Publisher's Note: MDPI stays neutral with regard to jurisdictional claims in published maps and institutional affiliations.

© 2020 by the authors. Submitted for possible open access publication under the terms and conditions of the Creative Commons Attribution (CC BY) license (http://creativecommons.org/licenses/by/4.0/).
